# Impact of Melatonin Application in Winemaking on Phenolic Content, Tryptophan Metabolites, and Bioactivity of Red Wine †

**DOI:** 10.3390/antiox14050504

**Published:** 2025-04-23

**Authors:** Neda Đorđević, Nevena Todorović Vukotić, Ivana Perić, Otilija Keta, Vladana Petković, Snežana B. Pajović, Branislav Nastasijević

**Affiliations:** 1 Department of Molecular Biology and Endocrinology, “Vinča” Institute of Nuclear Sciences—National Institute of the Republic of Serbia, University of Belgrade, 11000 Belgrade, Serbia; nevenat@vin.bg.ac.rs (N.T.V.); ivanap@vin.bg.ac.rs (I.P.); otilijak@vin.bg.ac.rs (O.K.); vladana@vin.bg.ac.rs (V.P.); pajovic@vin.bg.ac.rs (S.B.P.); 2 Department of Physical Chemistry, “Vinča” Institute of Nuclear Sciences—National Institute of the Republic of Serbia, University of Belgrade, 11000 Belgrade, Serbia; branislav@vin.bg.ac.rs

**Keywords:** red wine, melatonin, antioxidant activity, phenolics, anthocyanins, tryptophan metabolites, cytotoxic activity

## Abstract

Global wine consumption drives the interest for high-quality wine with enhanced health benefits. Yeast-produced tryptophan metabolites, including melatonin, a potent antioxidant, emerged as promising agents for enhancing functional properties of food and beverages. This study represents the pioneering work addressing whether melatonin supplementation during vinification affects Moldova red wine quality. Total phenolic/flavonoid contents, DPPH, and FRAP assays were measured via spectrophotometry, anthocyanins, and tryptophan metabolites using UPLC-MS/MS and UPLC-FLD, as well as cytotoxicity with the MTT assay. Results showed that addition of melatonin during the winemaking process increased total phenolic/flavonoid content, as well as the antioxidant capacity evidenced by increased anti-DPPH radical activity. These effects might be due to the stimulation of phenolic compound biosynthesis, particularly anthocyanins malvidin-3-O-glucoside, peonidin-3-O-glucoside, and delphinidin 3-O-glucoside, which were found to be increased in the treated wine. Additionally, the study revealed that melatonin-enriched wine exhibited increased cytotoxicity against two cancer cell lines, HCT116 and PANC-1. Finally, melatonin supplementation enhanced the concentration of kynurenic acid, which, due to its cytoprotective and antioxidant properties, could further increase the health benefits of the resulting wine. These findings offer promising avenue for future research of melatonin-driven functional properties of wine and provide step forward to a natural product with added value.

## 1. Introduction

Wine, one of the oldest and most popular alcoholic beverages in the world, has a long-standing tradition rooted in human history. The art of winemaking, or vinification, represents one of the most established fermentation processes, passed down through generations as an essential cultural practice [1]. During red wine production, the must is fermented along with the grape skins and other grape parts. As a result, the majority of the grape’s bioactive compounds, particularly polyphenols, are transferred into the wine, significantly contributing to its distinct organoleptic properties. Over 100 polyphenol compounds, including both flavonoids and non-flavonoids, have been identified in red wines using chromatography-mass spectrometry and other advanced analytical techniques [2]. It is estimated that 1 L of young red wine contains approximately 1–5 g of polyphenols [3]. Dominant polyphenols in red wine are flavonoids, such as catechin and epicatechin, their oligomers and polymers known as proanthocyanidins or condensed tannins, anthocyanins like malvidin-3-O-glucoside (Glc) and cyanidine-3-O-Glc, and flavonols such as quercetin, myricetin, and kaempferol [4]. Red wine and its polyphenols have been widely studied regarding their antioxidant properties and health benefits, mainly in reducing the risk of developing cardiovascular diseases, diabetes, neurodegenerative disorders, and cancers [5,6,7,8,9]. Polyphenols have been considered the main antioxidants in wine for a long time. However, recent discoveries identified metabolites of aromatic amino acids produced by yeast, which engaged scientific attention due to their potential synergistic and additive effects with other antioxidants during the winemaking process [10,11,12]. Among these, particular attention is being provided to indolic compounds, such as melatonin (MEL) and serotonin (SER), which are products of tryptophan (TRP) catabolism.

In humans, SER and MEL are produced in the pineal gland, and they are secreted and distributed throughout the organism to exert specific functions. MEL in wines comes from grape cells and yeasts from their precursors, TRP and SER, at specific stages of the winemaking process [13]. The concentration of MEL varies across different grape varieties and wines, ranging from picograms to nanograms per milliliter, depending on the fermentation process [14]. These levels are sufficient to reach significant plasma concentrations through dietary intake, offering potential health benefits [14]. Recent research has explored MEL’s role in various areas, including vinification, due to its protective effects against oxidative stress [15,16,17]. As a potent antioxidant, MEL scavenges free radicals, potentially enhancing the antioxidant profile of MEL-enriched wine and reducing oxidative stress, which may counteract cancer development and progression [18]. MEL not only directly scavenges free radicals but also stimulates antioxidant enzymes, suppresses prooxidant enzymes, and reduces radical formation by improving mitochondrial function [19]. Additionally, MEL’s anti-inflammatory properties create a less favorable environment for cancer cells [20]. It also influences cellular signaling pathways involved in cell proliferation, apoptosis, and metastasis, promoting the death of cancer cells and inhibiting their growth and spread [21]. Beyond its endogenous antioxidant properties, MEL enhances the effects of polyphenols through a synergistic interaction with these antioxidants. It has also shown immunomodulatory and neuroprotective effects [19,22,23]. Previous research has indicated an increased content of total volatile compounds, along with significant differences in the composition of volatile profiles in wines produced from MEL-treated grapes compared to those made from untreated grapes. These differences have been associated with enhanced fruity, spicy, and sweet sensory characteristics [24]. SER, which is an intermediate in the MEL biosynthesis pathway, plays a crucial role in maintaining cardiovascular, respiratory, thermoregulatory, and immune health. It is also involved in regulating feeding behavior, circadian rhythms (the sleep–wake cycle), and pain sensitivity, and it is implicated in the development of several neurological disorders, such as Parkinson’s and Alzheimer’s diseases [25]. Both MEL and SER contribute to the detoxification of free radicals, protecting essential molecules from the harmful effects of oxidative stress. Kynurenic acid (KYNA) is another TRP metabolite synthesized via the kynurenine (KYN) pathway in bacteria, fungi, plants, and animals. It is also produced by yeast during fermentation. Known for its neuroprotective, anti-inflammatory, antioxidative, and metabolic properties, KYNA has been the focus of scientific research in recent years [26,27].

Some grapevine varieties require frequent spraying, up to 10 times a year or more in rainy seasons, prompting many winegrowers to shift towards varieties that need little to no spraying [28]. Over recent decades, numerous such varieties with varying maturation times and agrobiological traits have been developed globally [29,30]. These varieties are now being tested in local vineyards, with some showing excellent results and thriving with minimal pesticide use or biological protection [31]. Moldova, a black grape variety bred in Moldova, is widely grown in Serbia, Macedonia, and across the Balkans. The variety is listed in the Vitis International Variety Catalogue (VIVC) with the variety number 7896, and its cultivar name is Moldova [32]. It is resistant to phylloxera and fungal diseases like downy mildew and gray mold, making it ideal for organic farming with minimal protection.

As a recent study demonstrated, MEL synthesis and its supplementation enhance the stability of the fermentation process by preserving yeast cell viability and reducing oxidative damage [33]. MEL exerts protective effects on *Saccharomyces cerevisiae* wine strains during alcoholic fermentation by mitigating oxidative stress under mild ethanol stress conditions. Moreover, MEL-induced physiological changes in both yeast and grape cells, acting independently or synergistically, may significantly influence the final characteristics of the wine. In this context, a previous study has shown that the exogenous addition of MEL at the initial stages of red wine vinification promotes the biosynthesis of polyphe-nolic compounds, likely by stimulating the activity of key enzymes such as phenylalanine ammonia lyase (PAL) and cinnamate 4-hydroxylase (C4H), which play essential roles in this process [10]. Additionally, it has been demonstrated that the same treatment modulates the expression of specific genes involved in anthocyanin biosynthesis, particularly 3-glycosyltransferase (3GT). The findings further reveal that this intervention enhances the total phenolic content and increases the concentrations of specific phenolic compounds, such as resveratrol, quercetin, and cyanidin-3-Glc [10]. Furthermore, due to its strong antioxidant properties, MEL shows preservative-like effects under a range of storage conditions, potentially supporting the maintenance of wine quality throughout prolonged aging periods.

However, research in this area is still in its early stages, and so far, it has mainly focused on the in vitro antioxidant activity of wine. The effects of exogenously applied melatonin during vinification on the broader biological activity of the final product remain largely unexplored. Thus, in addition to further investigating the effect of MEL treatment on the phenolic content and antioxidant properties of Moldova red wine during the vinification process, this study primarily focuses on examining how MEL supplementation influences the cytotoxic effects of Moldova wine on HCT116 and PANC-1 cancer cell lines. It also aims to determine whether the addition of MEL during vinification alters the content of TRP metabolites in the wine due to their notable biological significance. This study represents the pioneering work addressing this topic and breaks new ground in understanding the potential health benefits of MEL-enhanced wine.

## 2. Materials and Methods

### 2.1. Reagents

Folin–Ciocalteu regens, Na_2_CO_3_, gallic acid, rutin, AlCl_3_, CH_3_COOK, 2,4,6-tripyridyl-s-triazine (TPTZ), FeCl_3_, 2,2-Diphenyl-1-picrylhydrazyl, L-tryptophan, serotonin hydrochloride, and kynurenic acid were purchased from Sigma Aldrich (St. Louis, MO, USA), melatonin and Trolox were purchased from Thermo Scientific Chemicals (Waltham, MA, USA), and acetonitrile and methanol (HPLC grade) were purchased from J.T. Baker (Deventer, The Netherlands), as well as formic acid and dimethyl sulfoxide (DMSO). Absolute ethanol (HPLC grade) was purchased from Fisher Chemicals (Loughborough, UK). Deionized water was obtained using the PuriteSelect Fusion system (Thame, UK). MRC-5, HTC116, and PANC-1 cell lines were obtained from American Type Culture Collection (ATCC, Rockville, MD, USA). RPMI-1640 medium, fetal bovine serum, penicillin/streptomycin, L-glutamine, and 3-(4,5-dimethylthiazol-2-yl)-2,5-diphenyltetrazolium bromide (MTT) were purchased from Sigma–Aldrich Chemie GmbH (Steinheim, Germany).

### 2.2. Wine Sampling

In this study, Moldova grapes from the 2023 vintage were used to produce wines for further analysis. The grapes were manually harvested at full maturity with soluble sugars of 220.7 ± 0.5 g L^−1^ and total acidity of 4.6 ± 0.08 g L^−1^. The red wines were obtained by microvinification according to the previously published protocol [10]. The grapes were sulfited with 50 mg L^−1^ SO_2_, destemmed by hand, and crushed. The must was subjected to the process of controlled fermentation in glass fermentation vessels. *Saccharomyces cerevisiae* Actiflore F 33 (20 g hL^−1^) was used to initiate fermentation in both untreated and MEL-treated wines, with two replicates in both cases. The fermentation took place at 26 °C. Traditional maceration (duration of maceration was 7 days) followed by racking was employed to obtain the wines. A treatment group was prepared by adding 500 µg of MEL to 1 kg of must at the pre-winemaking stage. After 3 months of aging, both control and MEL-treated wines were analysed to assess the effects of the treatment on the wine’s characteristics. At the end of fermentation, the wines were racked, bottled, and stored in controlled conditions of 8–10 °C.

### 2.3. Total Phenolic Content

The total phenolic content (TPC) in wine samples was determined using the colorimetric Folin–Ciocalteu (FC) procedure, adapted by Singleton in 1998 for wine analysis [34]. This method relies on the electron transfer from phenolic compounds to a complex mixture of phosphotungstic acid (H_3_PW_12_O_40_) and phosphomolybdic acid (H_3_PMo_12_O_40_) in a basic medium. The reduction produces blue-colored tungsten oxide (W_8_O_23_) and molybdenum oxide (Mo_8_O_23_), which absorb light in the visible range between 715 and 750 nm. The intensity of light absorption at this wavelength is directly proportional to the concentration of total phenolics in the solution. The wines were diluted with deionized water to achieve an absorbance between 0.2 and 0.7 after reacting with the FC reagent. A 200 μL aliquot of the diluted wine solution was mixed with 1000 μL of FC reagent, previously diluted with distilled water at a 1:10 ratio. The mixture was left to stand for 6 min in the dark, after which 800 μL of a 7.5% Na_2_CO_3_ solution was added and vortexed to mix. The reaction was allowed to proceed for 2 h in the dark. Absorbance was then measured at 760 nm using a GBC Cintra 40 spectrophotometer (Boeckel & Co., Hamburg, Germany). The same procedure was followed for gallic acid standards at concentrations of 100, 200, 500, 1000, and 1500 mg L^−1^. All measurements were done in triplicate. The total phenolic content in the wines was calculated from the gallic acid standard curve and expressed as gallic acid equivalents (mg GAE L^−1^).

### 2.4. Total Flavonoid Content

The total flavonoid content (TFC) in wine samples was determined using a spectrophotometric method with aluminum(III) chloride (AlCl_3_) as the reagent [35]. This colorimetric method is based on the formation of stable complexes between AlCl_3_ and the C-4 keto group, as well as the C-3 or C-5 hydroxyl group of flavones and flavonols. Additionally, AlCl_3_ forms complexes with ortho-dihydroxyl (catechol) groups in the A or B ring of flavonoids. The absorbance of the light by the formed complexes was monitored at 410–440 nm.

For this test, wine samples were diluted with deionized water in the same manner as in the FC test. A reaction mixture was prepared by combining 1.0 mL of the wine sample, 50 µL of a 10% AlCl_3_ solution, 50 µL of a 1 M CH_3_COOK solution, and 1400 µL of deionized water. After allowing the reaction to proceed for 30 min, the absorbance of the mixture was measured at 410 nm using a GBC Cintra 40 spectrophotometer (Boeckel & Co., Hamburg, Germany). The same procedure was followed for five concentrations of a rutin standard solution (20, 25, 50, 100, and 200 mg L^−1^). The total flavonoid content in the wines was expressed in rutin equivalents (mg RTE L^−1^), calculated from the rutin standard curve. All measurements were performed in triplicate.

### 2.5. Anti-DPPH Radical Activity

The antiradical activity of the wine samples was evaluated using the DPPH assay, which measures antioxidant capacity based on the neutralization of DPPH radicals. The test tracks the reduction of the purple DPPH radical to its yellow reduced form, DPPH-H. A modified method from Blois was applied [36,37]. The wine samples were diluted with deionized water to ensure that the absorbance of the solution, after reacting with the DPPH radical, fell within the range of 0.2–0.7. A 100 μL diluted wine solution was mixed with 500 μL of a methanolic solution of DPPH (0.04 mg L^−1^) and allowed to react in the dark at room temperature for 30 min. Absorbance was measured at 517 nm using a GBC Cintra 40 spectrophotometer (Boeckel & Co., Hamburg, Germany). The wine’s ability to neutralize the DPPH radical was calculated using the equation:

DPPH (%) = [1 − A_x_/A_0_] × 100

where A_0_ is the absorbance of the control (with deionized water instead of wine) and A_x_ is the absorbance of the remaining DPPH radical after reacting with the wine solution. Each wine sample was tested at four different dilutions, and each dilution was analysed in triplicate. The results were expressed as EC50^−1^ values, indicating the reciprocal dilution of the wine sample that can scavenge 50% of the DPPH radical, derived from a graph showing the percentage of DPPH radical neutralization as a function of wine dilution.

### 2.6. FRAP Assay

The FRAP (Ferric Reducing Antioxidant Power) test measures the ability of wine samples to reduce the Fe(III)-TPTZ (2,4,6-tripyridyl-s-triazine) complex to the Fe(II) complex in an acidic medium, resulting in an intense blue color. The absorbance of this complex is measured at 593 nm. The method follows the original protocol by Benzie and Strain, with some modifications [38]. To prepare the working FRAP solution, 75 mL of 0.3 M acetate buffer at pH 3.6, 7.5 mL of 10 mM TPTZ, and 7.5 mL of 20 mM FeCl_3_ were mixed together. This solution was pre-incubated at 37 °C. The reaction mixture was created by combining 950 μL of the FRAP reagent with 50 μL of wine sample diluted 25 times. After mixing, the solution was incubated at 37 °C for 10 min, after which the absorbance was measured at 593 nm using a GBC Cintra 40 spectrophotometer (Boeckel & Co., Hamburg, Germany). The reducing power of the wine samples was expressed in Trolox equivalents (TE) (mmol Trolox L^−1^ wine).

### 2.7. UPLC Analysis

#### 2.7.1. Qualitative Analysis of Wine Samples by UPLC with Tandem Mass Spectrometry Detector

Waters ACQUITY Ultra Performance Liquid Chromatography (UPLC) system coupled with a Waters Mircomass Quattro micro API mass spectrometer (Waters, Milford, MA, USA) was used for qualitative analysis of wine samples. Data acquisition was performed using MassLynx software, version 4.1., in Multi Reaction Monitoring (MRM) mode, in both ESI positive and negative polarity. For the determination of anthocyanins in wine samples, linear gradient elution was applied on an ACQUITY UPLCTM BEH C18 column (1.7 μm, 100 mm × 2.1 mm). Mobile phase A contained 5% formic acid in water, and mobile phase B was 100% acetonitrile. Elution was performed at a flow rate of 0.25 mL min^−1^, column T = 40 °C, and injection volume of 5 μL. Considering the variety of compounds belonging to different classes of compounds, the gradient lasted 22 min and started with 95% A, then decreased to 80% A from 1–5 min, held 2 min at 80% A, then decreased to 70% from 7–10 min and held 2 min at 70% A. In the next 6 min, A gradually decreased to 10%, and in the next 4 min, it returned to 95%. Qualitative analysis of anthocyanins using the aforementioned method was performed after solid-phase extraction (SPE) of wine samples. Before UPLC analysis, samples were filtered through 0.22 μm nylon filters (Phenomenex, Torrance, CA, USA) and diluted five times using a mixture of deionized water:acetonitirile (90:10, *v*/*v*) with 0.1% formic acid. The elucidation of constituents in the wine samples was conducted using chemical standards. Identification was performed by comparing the retention time of the standard’s peak with corresponding peaks found in the wine samples and according to the characteristic MRM transitions (TQD) of selected constituents [39]. After optimization of ionization parameters, the following instrument settings were applied: capillary voltage 2.1 kV, LM1/HM1 resolution 13.0/14.6, LM2/HM2 resolution 14.0/14.8, RF lens voltage 0.2 V, extractor voltage 2 V, multiplier voltage 500 V, desolvation temperature 295 °C, desolvaton gas flow 700 L h^−1^, and cone gas flow 50 L h^−1^. The obtained results were expressed as the content ratio, which represents the ratio of the peak areas obtained for a specific anthocyanin compound (malvidin-3-O-Glc, cyanidine-3-O-Glc, delphinidin-3-O-Glc, petunidin-3-O-Glc, and peonidin-3-O-Glc) according to the monitored MRM transition.

#### 2.7.2. Solid-Phase Extraction Procedure

Before extraction, Strata C18-E (500 mg 5 mL^−1^, Phenomenex) cartridges were conditioned with 4 mL of methanol and washed with 4 mL of deionized water. Then, 4 mL of wine samples were loaded onto the cartridges, washed with 2 mL of deionized water, and eluted with 2 mL of methanol. The eluents were evaporated under the gentle stream of nitrogen gas and redissolved in 800 μL of methanol. After dilution with deionized water (1:2), the wine samples were analysed by UPLC-TQD.

#### 2.7.3. Quantitative Determination of Tryptophan Metabolites Using UPLC with Fluorescent Detector

Waters ACQUITY Ultra Performance Liquid Chromatography (UPLC) system coupled with an FLD detector 2475 was used for the quantitative determination of TRP and its metabolites: SER, MEL, and KYNA in wine samples prepared with and without the addition of MEL. Data acquisition was performed using Empower software 2.0. Quantitative analysis of TRP and its metabolites was done by direct injection of wine samples without SPE clean-up procedure. Before analysis, samples were filtered through 0.22 μm nylon filters (Phenomenex, Torrance, CA, USA) and separated on an ACQUITY UPLCTM BEH C18 column (1.7 μm, 100 mm × 2.1 mm) using linear gradient elution, with mobile phase A consisting of 0.1% formic acid in water and mobile phase B consisting of 0.1% formic acid in acetonitrile. The gradient started with 99% A and decreased to 40% within 6 min, then in the next 3 min, from 40 to 0% A, it returned to 99% A from 9–12 min. The flow rate was 0.25 mL min^−1^, column T was 40 °C, and the injection volume was 5 μL. Fluorescence detection was monitored at three channels for determination of SER and MEL at excitation/emission wavelengths λex = 297 nm and λem = 344 nm, for TRP at λex = 280 nm and λem = 350 nm, and for KYNA at λex = 330 nm and λem = 390 nm [40,41]. Standard solutions of TRP and its metabolites were prepared by mixing different volumes of stock solutions whose concentrations were 2 mg mL^−1^ (in deionized water) for SER, 2.5 mg mL^−1^ (in DMSO) for KYNA, 5 mg mL^−1^ for TRP (in deionized water), and 5 mg mL^−1^ for MEL dissolved in ethanol. The working mixtures of standard solutions used for the calibration curves were made by serial dilution of stock solution with deionized water: acetonitirile mixture (90:10%, *v*/*v*, with 0.1% formic acid) in the concentration range from 0.05 μg mL^−1^–0.5 μg mL^−1^.

### 2.8. Cytotoxic Activity

For the analysis of the cytotoxic effects of Moldova red wine, three human cell lines were used: healthy fibroblast cells MRC-5, colorectal carcinoma cells HCT116, and pancreatic carcinoma cells PANC-1. Cells were cultured in RPMI-1640 medium supplemented with 10% fetal bovine serum, penicillin/streptomycin antibiotic mixture, and L-glutamine. The culture was maintained in a humidified atmosphere with 5% CO_2_ at 37 °C. For the experiment, the cells were seeded into 96-well microtiter plates (Sarstedt, Newton, NC, USA) at a density of 2 × 10^3^ cells per well. During their exponential growth phase, the cells were treated with two concentrations of the analysed Moldova wines.

To assess the cytotoxic activity of the wines on the MRC-5, HCT116, and PANC-1 cell lines, the MTT assay (3-(4,5-dimethylthiazol-2-yl)-2,5-diphenyltetrazolium bromide) was employed [42]. After 48 h of exposure to the wines at concentrations of 2.5% and 5%, the absorbance was measured at 550 nm using an ELISA plate reader (Wallac, Victor2 1420 Multilabel Counter, PerkinElmer, Turku, Finland). The results were expressed as a percentage of the control, which represented 100% cell survival.

### 2.9. Statistical Analysis

The statistical analysis was conducted using Microsoft Office Excel 2019. Data are presented as mean ± standard deviation (SD). The statistical significance of the measurements was determined using a *t*-test, with significance levels indicated as follows: * *p* < 0.05, ** *p* < 0.01, and *** *p* < 0.001.

## 3. Results and Discussion

### 3.1. Total Phenolic/Flavonoid Content and Antioxidant Activity in Moldova Wine

The values obtained for TPC and TFC measurements (Table 1) showed that the TPC of Moldova wine treated with MEL was approximately 8% higher than that of the control wine sample (** *p* < 0.01). Similarly, the TFC in Moldova wine with added MEL was around 12% higher compared to the untreated wine (** *p* < 0.01). These results are in line with the study of Eremia et al., which observed an increase in the TPC of wine treated with MEL: a 9% increase in Cabernet Sauvignon wine, and an even greater increase of 24% in Fateasca Neagra wine [10]. This study demonstrated an increase in the concentration of specific phenolic compounds in wines treated with MEL, including resveratrol, quercetin, myricetin, trans-cinnamic acid, and *p*-coumaric acid. In addition, a slight elevation was observed in the levels of rutin and quercitrin. The next study, which investigated the effect of spraying Moldova grapes with an MEL solution during the ripening process on the chemical characteristics of the resulting wine, also showed an increase in the TPC and TFC by approximately 18.2% and 26.2%, respectively [43]. The same study also showed an increase in the proanthocyanidin content in Moldova wine obtained after treating the grapes with MEL by 4.47%. MEL added during vinification significantly boosts the phenolic content of the resulting wine, likely through its effects on the polyphenol biosynthetic pathway via the shikimate pathway and/or phenylpropanoid metabolism in both grapes and wine. Previous studies have shown that MEL treatment of grape berries during the pre-season period enhances the activity and expression of key enzymes for flavonoid synthesis. First of all, MEL enhances the activity of the enzyme PAL, which catalyzes phenylalanine deamination in the polyphenol biosynthesis pathway [44]. It also upregulates the genes involved in flavonoid biosynthesis, including 4-coumaroyl-CoA synthase (4CL), chalcone synthase (CHS), flavonoid 3′-hydroxylase (F3H), O-methyltransferases (OMT), flavonol synthase (FLS), flavonoid-3-O-glucosyltransferase (UDPG), and flavonoid 3′,5′-methyltransferase (AOMT) [45]. Thus, MEL treatment increased levels of cyanin-3-O-Glc, peonidin derivatives, and two flavanols, (+)-catechin and (−)-epicatechin, in both grapes and wine [46]. Additionally, MEL application on grapes at veraison was found to improve berry coloration by increasing transcript levels of anthocyanin biosynthesis genes [47]. The TPC content in Moldova wines detected in this study is less than the values obtained for Vranac, Merlot, and Cabernet Sauvignon commercial wines [48]. However, the TPC observed is consistent with findings by Mitrevska et al. and aligns globally with the typical TPC levels found in red wines, which generally range from 1 to 5 g L^−1^ [3,49].

**Figure 1:**
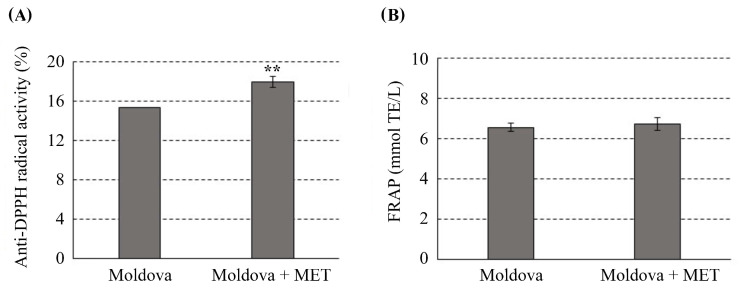
(**A**) Anti-DPPH radical activity and (**B**) FRAP assay values obtained for analysed wines; TE—Trolox equivalents; ** *p* < 0.01, *t*-test [50].

The antiradical activity of the wine samples, evaluated using the DPPH assay, as well as results obtained in the FRAP assay, is presented in Figure 1. The DPPH assay is one of the most widely used methods to assess antioxidant capacity, relying on electron transfer to reduce an oxidant. As shown in Figure 1A, the use of MEL significantly enhanced the ability to scavenge DPPH free radicals with a near-20% increase. This finding aligns with a recent study on Feteasca Neagra wine, where the addition of 500 μg of MEL per 1 kg of must during winemaking led to a 14% increase in anti-DPPH activity [10]. Furthermore, the study of Xu et al. showed a 32.8% increase in the DPPH assay analysing Moldova wine obtained from grapes pretreated with MEL [43].

The FRAP assay results showed no significant difference, with values of 6.57 ± 0.19 mmol TE L^−1^ for the untreated Moldova wine and 6.74 ± 0.31 mmol TE L^−1^ for the Moldova wine with added MEL (Figure 1B). Reducing the capacity of wine is an important indicator of its potential antioxidant activity. Polyphenols can reduce Fe^3+^ to Fe^2+^ through a one-electron-transfer pathway, where they are oxidized to semiquinones. Additionally, phenolic compounds exhibit strong antioxidant properties by forming complexes with metal ions, particularly iron and copper, which can be toxic in excess. MEL is also known to bind with ions such as zinc, lead, copper, iron, aluminum, and cadmium. These interactions with metals depend on MEL concentration, and MEL can chelate both Fe^3+^ and Fe^2+^ ions [3]. This underscores the synergistic effects between MEL and the antioxidant components in wine, enhancing its overall antioxidant capacity. In this context, MEL also directly influences the degradation of polyphenols induced by metal-catalyzed oxidation through the Fenton reaction. By chelating metal ions, MEL helps prevent this oxidative degradation, thus contributing to the preservation of the wine’s antioxidant potential and phenolic composition during aging and storage [51].

**Figure 2:**
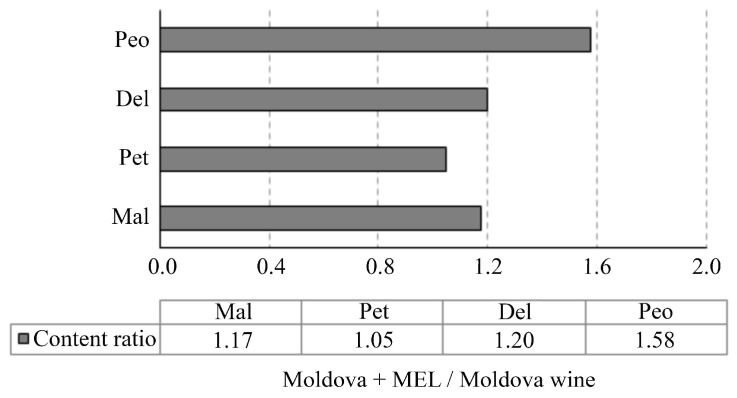
Content ratio of anthocyanin compounds of Moldova + MEL and Moldova wines; Mal—malvidine-3-O-Glc, Pet—petunidin-3-O-Glc, Del—delphinidin-3-O-Glc, Peo—peonidin-3-O-Glc.

The DPPH and FRAP assays indicated that adding MEL during the winemaking process leads to wine with a higher free-radical scavenging capacity and an equally effective reduction of antioxidant power. Although the exact mechanisms by which MEL enhances the antioxidant capacity of wine remain unclear, it is plausible that it plays a significant role in neutralizing reactive oxygen species (ROS) such as hydroxyl radicals, hydrogen peroxide, superoxide anions, singlet oxygen, peroxynitrite anion, nitric oxide, and hypochlorous acid. In addition to its direct antioxidant activity, MEL also indirectly boosts the body’s defense mechanisms by stimulating key antioxidant enzymes, including glutathione peroxidase (GPx), superoxide dismutase (SOD), glutathione reductase (GLR), and catalase (CAT), while simultaneously inhibiting pro-oxidant enzyme activity [52]. Namely, previous studies have shown that exogenously added melatonin can influence the modulation of redox balance during fruit storage. Specifically, MEL slows down enzymatic oxidation catalyzed by polyphenol oxidase (PPO) and peroxidase (POD), which prevents browning reactions and unwanted polymerization of phenolics. As a result, MEL contributes to the better preservation of both the color and structural integrity of phenolic compounds [53,54,55]. Furthermore, these studies revealed that exogenous MEL maintains higher activities of antioxidant enzymes such as SOD, GR, and CAT, which further help prevent oxidative processes in fruit, including the inhibition of polyphenol oxidation. It can be assumed that the effect of melatonin is similar in the fermentation process of wine. MEL also enhances the antioxidant capacity of wine by promoting the accumulation of polyphenols, which are positively correlated with the wine’s overall antioxidant properties [56,57]. Both direct and indirect antioxidant effects of MEL aid in minimizing oxidative damage in wine during fermentation by protecting phenolic and other sensitive compounds from oxidation, further reducing the formation of off-flavors and preventing wine color and flavor/aroma changes [58]. In line with the aforementioned results, this study demonstrated that MEL-enriched wine exhibited increased levels of total phenols and flavonoids, directly contributing to an enhanced radical-scavenging activity of the wine as a product. Additionally, higher levels of three anthocyanins, malvidine 3-O-Glc, delphinidine 3-O-Glc, and peonidine 3-O-Glc in the MEL-enriched wine (Figure 2) might contribute positively to the increased antioxidant capacity of the resulting wine. The fact that these compounds are described as main contributors to the in vivo antioxidant capacity of red wines supports this explanation [59].

### 3.2. Anthocyanins Profile Analysis

A qualitative analysis of wines prepared with and without the addition of MEL in the vinification process is shown in Table 2. Constituents found in wine samples belonging to the anthocyanin compounds were identified according to their characteristic MRM transitions.

**Figure 3:**
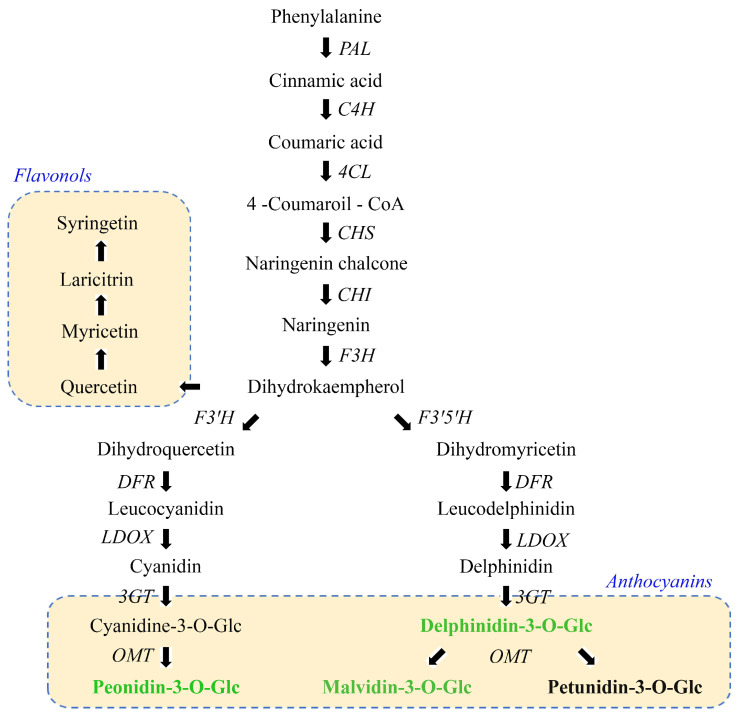
Schematic illustration of anthocyanidin biosynthesis. Compounds highlighted in bold were analysed in the current study, and those marked in green were found to be elevated in MEL-treated wine. PAL—phenylalanine ammonia lyase; C4H—cinnamate 4-hydroxylase; 4CL—4-coumaroyl CoA ligase; CHS—chalcone synthase; CHI—chalcone isomerase; F3H—flavanone 3-hydroxylase; F3′H—flavonoid 3′-hydroxylase; F3′5′H—flavonoid 3′5′-hydroxylase; DFR—dihydroflavonol 4-reductase; LDOX—leucoanthocyanidin dioxygenase; 3GT—3-glucosyltransferase; OMT—O-methyl trasferase.

Anthocyanins are synthesised in the phenylpropanoid pathway under the complex control of numerous regulatory genes at the transcriptional level (Figure 3). They are glycosides of anthocyanidins, and six different anthocyanidins are found in nature, i.e., pelargonidin, cyanidin, delphinidin, peonidin, petunidin, and malvidin. In grapes, the sugar moiety is usually glucose at the C3 position. The most important anthocyanins in wines are the 3-O-monoglucosides of cyanidin, peonidin, delphinidin, petunidin, and malvidin, which are formed by the action of the 3-glucosyltransferase (3-GT) enzymes [60]. The higher content of the malvidine-3-O-Glc, delphinidin-3-O-Glc, and peonidin-3-O-Glc in the treated wine (1.17, 1.20, and 1.58 times higher, respectively) indicated that MEL affects the enzymes included in anthocyanin synthesis, i.e., it enhances the gene expression associated with anthocyanin biosynthesis. As we previously mentioned, MEL enhances the activity of the PAL, an enzyme that catalyzes the first step in the polyphenol biosynthesis pathway [44]. Furthermore, MEL upregulates the genes for 4CL, CHS, and F3H enzymes, which are important for obtaining the dihydrokaempherol, one of the main precursors in the biosynthetic pathway of anthocyanins. Through subsequent reactions, dihydrokaempherol undergoes a reaction to leucocyanidin, the compound that serves as precursor for catechins but also for cyanidin-3-O-Glc and peonidin-3-O-Glc due to the action of the 3-GT enzyme. The increased content of peonidin-3-O-Glc in MEL-treated wine, as well as the content of cyanidin-O-Glc being below the detection limit in the wine (potentially because this anthocyanin is the precursor of peonidin 3-O-Glc), may indicate that MEL further stimulates and shifts the process towards the biosynthesis of peonidin-3-O-Glc through the action of OMT. This enzyme catalyzes two sequential methylation reactions, utilizing S-adenosylmethionine as the methyl donor. This is in line with a previous study by Yang et al. that showed that exogenous MEL increases the OMT gene-expression level [45]. The RT-qPCR results of that study also showed that 4CL, CHS, F3′H, OMT, FLS, UDPG, and AOMT genes involved in flavonoid biosynthesis were more expressed, in different degrees, under MEL treatment [45].

Petunidin-3-O-Glc and malvidin-3-O-Glc are synthesized from the same precursor delphinidin-3-O-Glc, again through the action of the OMT. Our results show that MEL favors the formation of malvidin-3-O-Glc, judging by the rise of malvidin-3-O-Glc and the unchanged levels of petunidin-3-O-Glc in MEL-enriched wine. The intensity of signals obtained from HPLC analysis showed that malvidin-3-O-Glc is the most abundant monomeric anthocyanin in the obtained wines, as expected in young red wines [61]. Results of this study are in line with previously published results, which showed that MEL spraying of grapes induced higher transcript abundance of anthocyanin biosynthesis-related genes and transcription factors [47]. The same assumption was made in the study by Eremia et al. [10]. Considering all factors, it can be concluded that exogenously applied MEL results in increased production and/or accumulation of anthocyanins in wines, preferentially due to MET-induced changes in expression and/or activation of specific anthocyanin-related proteins, which remains to be further clarified.

### 3.3. Tryptophan Metabolites Analysis

**Figure 4:**
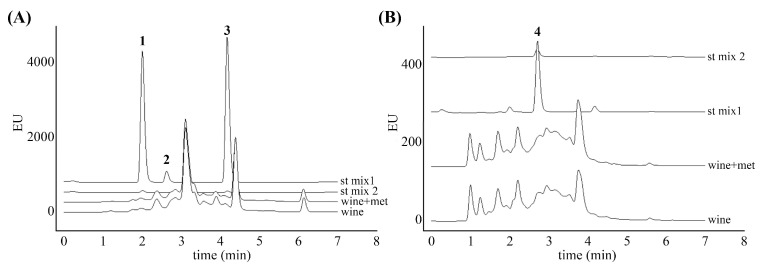
(**A**) Comparison of chromatograms of wines and standard mixtures of TRP and its metabolites (SER—1, TRP—2, and MEL—3), with concentrations 0.025 μg mL^−1^ (st mix 2) and 0.1 μg mL^−1^ (st mix 1). Chromatograms were obtained using UPLC with an FLD detector at ex/em = 297/344 nm; (**B**) chromatograms of KYNA in wines and standard mixtures were obtained at ex/em = 330/390 nm.

Quantitative determination of TRP metabolites was performed using a UPLC-FLD detector. As previously noted, FLD chromatograms were recorded using three channels, and the channels with the most intense fluorescence from each metabolite were chosen to determine individual metabolites quantitatively. UPLC-FLD chromatograms of wine samples and mixtures of standards of TRP and its metabolites are shown in Figure 4. Under previously described UPLC conditions, recorded retention times of analysed metabolite standards were 1.99 min for SER, 2.63 min for TRP, 2.72 min for KYNA, and 4.17 min for MEL. The presence of SER, KYNA, and MEL in the wine samples was confirmed by the overlap of their chromatographic peaks with those of the corresponding reference standards. The signal for TRP was not detected in wine samples, i.e., it was less than LOD (0.012 µg mL^−1^).

Contents of the analysed metabolites in wine samples are shown in Table 3.

**Figure 5:**
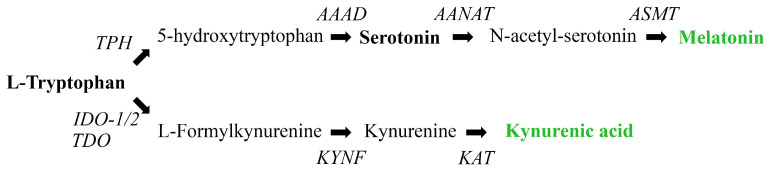
Schematic illustration of L-TRP conversion to MEL and KYNA. Compounds highlighted in bold were analysed in the current study, and those marked in green were found to be elevated in MEL-treated wine. TPH—tryptophan hydroxylase, AAAD—aromatic amino acid decarboxylase, AANAT—serotonin-N-acetyl transferase, ASMT—hydroxylindole-O-methyltransferase, IDO—indole-2,3-dioxygenase, TDO—tryptophan-dioxygenase, KYNF—kynurenine formamidase, KAT—kynurenine transaminase.

Grapes must contain a variety of nitrogen sources, with the most significant being amino acids, ammonium ions, and small peptides. Nitrogen impacts yeast cells in two key ways: it influences biomass production during fermentation and affects the fermentation rate [62]. The MEL biosynthesis pathway in yeasts appears to closely resemble the synthetic route and enzyme mechanisms described in vertebrates [63]. This process involves four enzymes that play a role in the straightforward conversion of TRP to SER and N-acetylserotonin intermediates, ultimately leading to the production of MEL (Figure 5). The obtained results in this study showed a significant increase in the content of MEL and KYNA in the wine with added MEL compared to untreated wine (*** *p* < 0.001 and ** *p* < 0.01, respectively) (Table 3). TRP was not detected in either of the analysed wines, indicating that its concentration was below the limit of detection. The causality may be attributed to its increased consumption, which likely promotes the production of its metabolites. The results indicated a three-fold increase in MEL concentration, which can be attributed to MEL added during the vinification process. This result is in line with previously published data showing that exogenous MEL treatment of some fruits and the resulting red wine promoted endogenous MEL biosynthesis [43,63,64]. However, a limitation of the current study is the inability to distinguish between MEL from external (added during vinification) and internal sources. Future studies should consider the use of isotopically labeled MEL standards in wine treatments to clearly differentiate between these sources. Nonetheless, based on the lack of observed changes in SER levels, we hypothesize that the MEL increment is exclusively due to external sources. It should also be noted that the fermentation process can produce by-products structurally similar to MEL, such as tryptophan ethyl ester, which are frequently misidentified as MEL [65,66,67]. Nonetheless, the study by Fracassetti et al. demonstrated that, despite its structural resemblance, tryptophan ethyl ester displays a sufficiently different retention time compared to MEL when analyzed using the HPLC-FL method, as applied in the present study [41], thereby supporting our conclusions obtained by FLD. Future research should aim to establish a comprehensive metabolomic profile of wine in order to gain deeper insight into the presence of different MEL isomers and related compounds, including tryptophan ethyl ester, that may contribute to the wine’s overall biological activity.

On the other hand, the 15% increase in KYNA content in wine with added MEL was noticed in this study (Table 3). L-TRP is processed through various pathways: 90–97% lead to the degradation of the indole ring, resulting in the formation of KYN and its derivatives, while 3–10% preserve the indole ring and generate chemical messengers from the indolamine family, including SER and MEL [68]. The KYN pathway is present not only in humans but also in yeasts [69]. KYNA, a cytoprotective metabolite, originates from the KYN pathway. Dei Cas et al. highlighted the dominance of metabolites from the KYN pathway, noting that the metabolism of TRP in *Saccharomyces cerevisiae* EC1118 is skewed in this direction. In that study, UPLC-FLD analysis revealed that KYNA, a molecule with neuroprotective and antioxidant properties, is synthesized in higher concentrations [68]. The levels of KYNA that we observed align with those reported in other studies [52,69]. In addition to the fact that the KYN pathway is dominant in TRP metabolism, the observed increase in KYNA levels may be attributed to a disruption in the balance of the KYN-SER pathways, potentially driven by elevated MEL concentrations. Recent studies indicate that MEL treatment leads to a downregulation of arylalkylamine N-acetyltransferase (AANAT), an enzyme responsible for converting SER to MEL, while simultaneously upregulating the expression of indole-2,3-dioxygenase 1 (IDO-1), a key rate-limiting enzyme in the conversion of TRP to KYNA [70]. This shift may provide an explanation for the increased KYNA level observed in MEL-treated wine. Since the primary physiological activity of grape cells typically ends before the ripening process, it can be hypothesized that MEL supplementation during fermentation primarily influences the physiological processes of yeast cells. As previous studies have demonstrated, the increased stability of yeast cells under elevated MEL conditions is attributed to enhanced tolerance to high ethanol concentrations, which results in reduced cell mortality [33]. Consequently, the elevated levels of TRP metabolites may reflect these changes associated with improved yeast survival. Additionally, as earlier studies indicate, MEL supplementation promotes endogenous MEL synthesis, potentially contributing to its increased concentration. Since the activity of enzymes within the TRP and KYN pathways is particularly sensitive to oxidative damage, it is plausible that elevated MEL levels could enhance the biosynthesis of downstream metabolites, such as SER, MEL, and related derivatives, by modulating oxidative stress during fermentation.

### 3.4. Cytotoxic Effects of Moldova Wines

**Figure 6:**
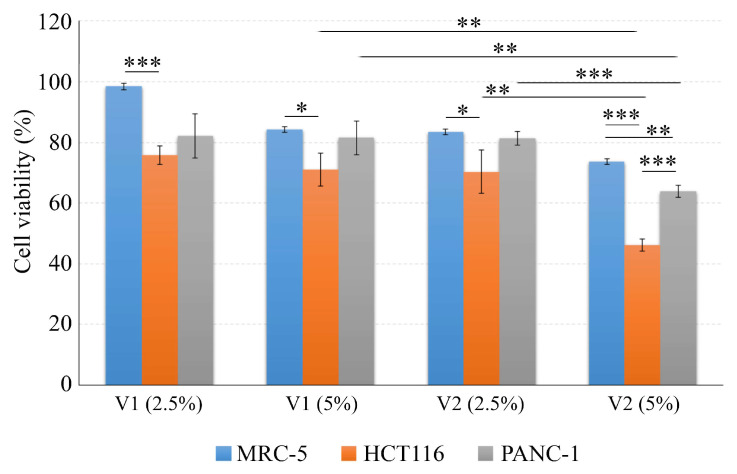
Cell viability after treatment with different wine concentrations (2.5% and 5%) of Moldova wine without added MEL in vinification process (V1) and Moldova wine with added MEL in vinification process (V2). MRC-1—healthy fibroblast cells, HCT116—colorectal carcinoma cell line and PANC-1—pancreatic carcinoma cell line. * *p* < 0.05, ** *p* < 0.01, and *** *p* < 0.001, *t*-test.

The cell viability in the presence of 2.5 and 5% Moldova wines indicated that the untreated Moldova wine and Moldova wine with added MEL had the lowest cytotoxic effect on healthy MRC-5 cells, ranging between 98.5 and 73.7% compared to untreated cells (Figure 6). Conversely, the cytotoxic impact on the two cancer cell lines demonstrated higher percentages, reaching 75.9% to 46.2% on HCT116 and 82.1% to 63.9% on PANC-1 cells. Specifically, both the 2.5 and 5% concentrations of untreated wine and wine with added MEL exhibited significantly higher cytotoxic effects on HCT116 compared to MRC-5 cells (*** *p* < 0.001 and * *p* < 0.05, * *p* < 0.05 and *** *p* < 0.001, respectively). The same trend was observed for the 5% wine with added MEL, which showed a comparable impact on the PANC-1 cell line (** *p* < 0.01). Interestingly, HCT116 cells were more sensitive to the 5% wine with added MEL than PANC-1 cells (*** *p* < 0.001). Additionally, a 5% concentration of wine with added MEL during the fermentation process demonstrated a significantly greater anti-proliferative effect on both cancer cell lines compared to a 5% concentration of untreated wine (** *p* <0.01). As the concentration of tested wines with MEL increased from 2.5% to 5%, a corresponding increase in cytotoxicity was observed on both HCT116 and PANC-1 cells (** *p* < 0.01 and *** *p* < 0.001, respectively), suggesting a dose-dependent relationship between the wine concentration and cytotoxicity.

MEL itself exerts antiproliferative, antimetastatic, and cytotoxic effects on different types of human malignancies, including colorectal and pancreatic cancer. Ji et al. revealed that MEL influences the cell cycle in colorectal cancer by regulating the miR-34a/449a cluster [71]. Additionally, both in vitro and in vivo studies demonstrated that MEL enhances the sensitivity of human colorectal cancer cells to gamma-ray ionizing radiation [72]. MEL also has the potential to prevent the progression of pancreatitis into pancreatic cancer [73]. Additionally, in pancreatic cancer, MEL induces apoptosis through its effects as an inhibitor of inflammation, a modulator of oxidative stress, a VEGF blocker, and an inhibitor of heat-shock proteins (HSPs) [74]. Considering that MEL does not cause any moderate-to-severe side effects, even at relatively high doses, it currently makes this molecule a prominent topic of research, particularly in cancer treatment studies [75]. It is known that phenolic compounds also induce apoptosis, cell cycle arrest, and increased ROS levels in cancer cells [76]. These compounds exert significant anticancer effects by targeting key molecular pathways. They lower the expression of a transcription factor involved in cytoprotective gene regulation, reduce p53 activation, and decrease Bcl-2 expression and mitochondrial membrane potential. Additionally, they suppress HIF-1α expression and promote apoptosis through the downregulation of p-Akt expression [76]. Since the MEL stimulates the activity of the biosynthetic enzymes and significantly alters the expression of specific genes involved in anthocyanin biosynthesis, it seems that MEL contributes to the protective cytotoxic ability of polyphenols in cancer cells by increasing their concentration in the treated wine [10].

## 4. Conclusions

Wine, being one of the most popular alcoholic beverages worldwide, is well-regarded for its beneficial effects on human health when consumed in recommended amounts. Consequently, there is a growing interest in improving its composition and quality. This study demonstrated that the addition of MEL during the winemaking process increased the phenolic content and antioxidant capacity of wine, presumably, in part, through the stimulation of phenolic compound biosynthesis, particularly anthocyanins. Most notably, MEL, through its potent antioxidant activity, likely mitigated oxidative stress and prevented the degradation of phenolic compounds, thereby contributing to the higher phenolic content observed in MEL-treated wine. Consequently, this enhancement in phenolic preservation was associated with an increased antioxidant capacity of wine. Additionally, the study revealed that MEL-enriched wine exhibited increased cytotoxic activity against two cancer cell lines, HCT116 and PANC-1. This effect can be linked to the higher concentration of MEL in the wine and the elevated levels of phenolic compounds, both of which contribute to its anticancer properties. The pioneering and most significant finding of this study is that MEL supplementation during winemaking enhances the synthesis of TRP metabolites, particularly KYNA (15% higher value), which, due to its cytoprotective and antioxidant properties, could further increase the health benefits of the resulting wine. Besides KYNA, the metabolism of aromatic amino acids in yeasts produces a wide range of molecules that may be important for both yeast regulation and human health. The neurohormonal and antioxidant activities of these compounds impart additional health benefits to wine. It should be noted that a lack of data regarding the antioxidant activity of MEL-enriched Moldova wine in cell culture, as well as effects following in vitro digestion, limits the physiological relevance of the presented findings. Further in vitro and in vivo studies are necessary to understand the underlying mechanisms, including signal pathways that are the basis of these processes. In future studies, it would also be of interest to investigate how the addition of MEL during the vinification process influences the organoleptic properties of the resulting wines. Taking everything into account, this study points toward a promising avenue for enhancing the functional properties of wine.

## Figures and Tables

**Table 1:** The total phenolic/flavonoid content of analysed wine samples.

Wine Sample	Total Phenolic Content (mg GAE L^−1^)	Total Flavonoid Content (mg RTE L^−1^)
Moldova	1045.0 ± 12.50	135.5 ± 3.36
Moldova + MEL	1132.5 ± 16.54 **	149.2 ± 4.30 **

GAE—gallic acid equivalents; RTE—ruthin equivalents; ** *p* < 0.01, *t*-test.

**Table 2:** The most abundant polyphenols and anthocyanins in wine samples (after SPE extractions) with their characteristic retention times, MRM transitions, and ionisation conditions.

Peak	Rt (min)	Ion Species	MRM *m*/*z* Transition	Cone Voltage (V)/Collision E (eV)	Putative Constituent
1	2.01	[M + H]^+^	465 > 303	40/20	Delphinidin-3-O-Glc
2	3.22	[M + H]^+^	479 > 317	40/20	Petunidin-3-O-Glc
3	3.70	[M + H]^+^	463 > 301	40/20	Peonidin-3-O-Glc
4	3.86	[M + H]^+^	493 > 331	40/20	Malvidine-3-O-Glc

**Table 3:** The content of Tryptophan and its metabolites (μg mL^−1^) in wine samples, determined by UPLC-FLD. Results are presented as mean ± SD; ** *p* < 0.01, *** *p* < 0.001 (*t*-test).

Sample	Serotonin	Tryptophan	Kynurenic Acid	Melatonin
Moldova	0.103 ± 0.005	<LOD	0.362 ± 0.012	0.035 ± 0.002
Moldova + melatonin	0.098 ± 0.013	<LOD	0.417 ± 0.013 **	0.104 ± 0.002 ***

LOD—Limit of detection (0.012 µg mL^−1^).

## Data Availability

The authors confirm that the data supporting the findings of this study are available within the article.
